# Sexual Dimorphism in the Relationship between Body Composition and Insulin Resistance in Older Adults

**DOI:** 10.1016/j.cdnut.2026.107707

**Published:** 2026-04-25

**Authors:** Dachuan Zhang, Sri Lakshmi S Devarakonda, Reilly A Roberts, Frank L Greenway, Candida J Rebello

**Affiliations:** 1 Biostatistics, Pennington Biomedical Research Center, Baton Rouge, LA, United States; 2 Metabolism and Body Composition, Pennington Biomedical Research Center, Baton Rouge, LA, United States; 3 Nutrition and Chronic Disease, Pennington Biomedical Research Center, Baton Rouge, LA, United States; 4 Clinical Trials Unit, Pennington Biomedical Research Center, Baton Rouge, LA, United States

**Keywords:** obesity, insulin resistance, body composition, sexual dimorphism, older adults

## Abstract

**Background:**

Epidemiological studies show an inconsistent relationship between lean mass and insulin resistance.

**Objectives:**

This study aimed to investigate the association between lean mass and insulin resistance in adults aged ∼60 to 80 y with and without obesity.

**Methods:**

We analyzed data from the Pennington/Louisiana Nutrition and Obesity Research Center (NORC) biorepository and NHANES. Lean mass was represented as total lean mass, along with total lean mass and appendicular lean mass (ALM) adjusted for body weight (% lean mass and % ALM), and total lean mass and ALM adjusted for height (lean mass/ht^2^ and ALM/ht^2^). We used partial correlation analyses to examine the relationship between insulin resistance (HOMA-IR) and body composition, and linear regression models to test for sex-by-body composition interactions.

**Results:**

In the NORC cohort with obesity, HOMA-IR positively correlated with total lean mass (*r* = 0.27, *P* = 0.005), lean mass/ht^2^ (*r* = 0.30, *P* = 0.002), and ALM/ht^2^ (*r* = 0.28, *P* = 0.004), only in females. In the NHANES cohort, HOMA-IR positively correlated with total lean mass (*r* = 0.23, *P* < 0.001), lean mass/ht^2^ (*r* = 0.20, *P* < 0.001), and ALM/ht^2^ (*r* = 0.14, *P* = 0.004) in females with obesity, and without obesity [total lean mass (*r* = 0.14, *P* = 0.001); lean mass/ht^2^ (*r* = 0.18, *P* < 0.001); and ALM/ht^2^ (*r* = 0.12, *P* = 0.005)]. In males, percent fat mass positively correlated with HOMA-IR in the NORC cohort with obesity (*r* = 0.27, *P* = 0.015) and in the NHANES cohort with obesity (*r* = 0.12, *P* = 0.043) and without obesity (*r* = 0.11, *P* = 0.005). Tests for interaction confirmed significant sex differences in the obesity cohorts for ALM/ht^2^ (NORC: *P* = 0.011), % fat mass (NORC: *P* = 0.016; NHANES: *P* = 0.002), and total lean mass (NHANES: *P* = 0.001). In the NHANES nonobesity cohort, significant interactions were observed for lean mass/ht^2^ (*P* = 0.001) and ALM/ht^2^ (*P* = 0.005).

**Conclusions:**

The relationship between body composition and insulin resistance displays sexual dimorphism. Although lean mass positively correlates with insulin resistance in females, in males, fat mass appears to be the dominant influence on insulin resistance.

## Introduction

Skeletal muscle is the most abundant tissue in the human body, representing 40% of body weight, and contributes to the majority of glucose uptake in response to insulin stimulation. Enhanced muscle mass, as observed in bodybuilders, is associated with improved blood glucose control and supports the view that enlargement of fat-free mass through training reduces the risk for diseases of impaired glucose metabolism. Given these findings, it is reasonable to assume that greater fat-free mass is associated with improved glucose homeostasis [1,2]. Some epidemiologic studies observed a negative association of lean mass with measures of glucose homeostasis consistent with the assumption that it is beneficial for glucose control [3,4]. However, other studies show a positive correlation or no correlation of lean mass with measures of insulin resistance [[5], [6], [7], [8], [9], [10], [11]].

The inconsistent results have been attributed, in part, to the representation of lean mass. When lean mass is expressed as a proportion of body weight, it could be that the relationship is influenced by the proportion of fat mass represented in body weight [12]. Nevertheless, controversy surrounds the relationship of lean mass with insulin resistance, particularly in older adults, where a high prevalence of obesity and insulin resistance puts them at high risk for impaired skeletal muscle function and frailty [[13], [14], [15]]. Similarly, this issue also arises in conditions like sarcopenic obesity, where high body fat and low muscle mass occur together, complicating interpretation of metabolic outcomes [16].

To address this shortcoming, the European Society for Clinical Nutrition and Metabolism and the European Association for the Study of Obesity launched an initiative to reach expert consensus on a definition and diagnostic criteria for sarcopenic obesity [16]. Because a relative reduction in skeletal muscle mass could result from increased body fat, the panel proposed a consensus framework emphasizing relative muscle mass. Fat-free mass adjusted for body weight was recommended because individuals with obesity may have higher absolute muscle mass relative to those without obesity, due to higher overall body mass and potentially higher muscle workload in activities of daily living. In addition, recommended diagnostic measures included appendicular lean mass (ALM) and fat mass, normalized to body weight. However, the panel acknowledged limitations of weight-based normalization and emphasized the need for further research to validate alternative adjustments (e.g., for height) in diagnosing sarcopenic obesity [16].

The biological pathways to sarcopenic obesity encompass age-related changes in body composition and insulin resistance [17]. Therefore, in light of the unresolved issue with lean mass normalization approaches, we investigated the association between insulin resistance and absolute values of lean mass, ALM, and fat mass, and each of these metrics normalized to weight or height, with special emphasis on lean mass. With the advent of incretin-based pharmacotherapy to treat obesity that causes rapid and significant loss of lean mass comparable to a decade or more of aging, the role of lean mass in metabolic outcomes assumes significance [18]. Because sex differences may play a role in the association between lean mass and insulin resistance, particularly in older adults (∼60–80 y) with obesity, we grouped the data by sex and obesity status.

## Methods

The Pennington/Louisiana Nutrition and Obesity Research Center (NORC) biorepository is a searchable archive of clinical data collected in human subjects research at the Pennington Biomedical Research Center since 1980 (https://my.pbrc.edu/NORC/NORCRepository/Landing). The National Health and Nutrition Examination Survey (NHANES) is a continuous, annual survey of the noninstitutionalized civilian resident population of the United States conducted by the Centers for Disease Control and Prevention. Oversampling is carried out for older Americans (aged ≥60), Mexican Americans, Blacks, and individuals at or <130% of the poverty level. The design specifications for the 1999 to 2006 survey, including the operational requirements, sample design, and estimation procedures, have been published (NHANES Questionnaires, Datasets, and Related Documentation).

Using data from the NORC biorepository and the 1999 to 2006 NHANES database, we performed a secondary analysis. From the NORC biorepository, we included adults aged 60 to 80 y who participated in prior clinical trials at Pennington Biomedical. To confirm and extend our findings, we then analyzed data from NHANES, selecting adults aged 59 to 79 y. We included participants with and without obesity, defined as BMI ≥30 kg/m^2^. Body composition was measured by dual X-ray absorptiometry (DXA). The HOMA-IR was used to estimate insulin resistance, which corresponds well but is not necessarily equivalent to estimates of insulin sensitivity derived from the hyperinsulinemic euglycemic clamp.

### Statistical analysis

Partial correlation analysis of HOMA-IR and body composition measures was performed separately for the NORC and the NHANES cohorts in participants with and without obesity. Race was accounted for in the NORC study to adjust for possible differences in metabolic profiles and body composition phenotypes. Additional confounding variables were available in the NHANES dataset, and adjustments were made for age, education level, smoking, arthritis and cancer status, and race.

In both datasets, HOMA-IR was considered the dependent variable. Total lean mass, adjusted for weight and height (% lean mass and lean mass/ht^2^, respectively), ALM, ALM adjusted for body weight and height (% ALM and ALM/ht^2^, respectively), and total fat mass adjusted for weight and height (% fat mass and fat mass/ht^2^, respectively) were treated as independent variables. Sex-stratified analyses were performed for potential sex-specific differences. In addition, linear regression models incorporating the same set of covariate adjustments described above were constructed to assess sex-by-body composition interactions. SAS version 9.4 (SAS Institute) was used for all statistical analyses. Statistical significance was determined using the *P* values associated with the partial correlation coefficients, with significance defined as *P* < 0.05.

## Results

Baseline characteristics of the two cohorts are presented in Table 1. Sex-by-body composition interactions are presented in Table 2. In the NORC cohort with obesity, significant interactions were observed for % lean mass (*P* = 0.020), ALM/ht^2^ (*P* = 0.011), and % fat mass (*P* = 0.016). These findings were largely confirmed in the NHANES cohort with obesity, which showed significant interactions for % lean mass (*P* = 0.002), % ALM (*P* = 0.043), % fat mass (*P* = 0.002), total lean mass (*P* = 0.001), and fat mass/ht^2^ (*P* = 0.047). In the NHANES cohort without obesity, significant interactions were observed for lean mass/ht^2^ (*P* = 0.001) and ALM/ht^2^ (*P* = 0.005). Significant interactions are presented in Supplementary Figures 1 to 10.

**TABLE 1:** Comparison of data from the NHANES and NORC databases

Variable	Measure	NHANES (*n* = 756) BMI ≥30 kg/m^2^	NORC (*n* = 188) BMI ≥30 kg/m^2^	NHANES (*n* = 1311) BMI <30 kg/m^2^	NORC (*n* = 113) BMI <30 kg/m^2^
Demographics	Age (y)^1^	67.0 ± 5.4	67.2 ± 5.1	68.2 ± 5.9	68.8 ± 5.8
	Female (%)	56.8	56.4	54.7	49.56
	Education level (%) (greater than or equal to high school)	59	—	—	—
Race	Non-Hispanic White (%)	48.3	73.9	52.6	85.0
	Non-Hispanic Black (%)	23.7	24.5	14.3	13.3
	Hispanic (%)	26.9	1.6	28.9	1.8
	Other (%)	1.2	0	4.1	0
Comorbidities	Current smoker (%)	2.9	—	4.9	—
	Diabetes (%)	9.3	—	4.7	—
	Arthritis (%)	16.8	—	13.0	—
	Cancer (%)	4.6	—	5.0	—
	Cardiovascular disease (%)	9.4	—	6.9	—
Weight status and blood measures	Weight (kg)^1^	95.7 ± 15.8	96.6 ± 13.2	71.3 ± 11.9	76.6 ± 11.6
	BMI (kg/m^2^)^1^	35 ± 4.4	34.7 ± 4.1	25.5 ± 2.9	26.7 ± 2
	Glucose (mg/dl)^1^	124.1 ± 48	120.3 ± 33.7	112.3 ± 41.7	107.8 ± 20
	Insulin (μU/mL)^1^	20 ± 19	18.2 ± 12.1	11.9 ± 19.1	13.1 ± 14.3
	% Lean mass	0.6 ± 0.1	0.6 ± 0.1	0.6 ± 0.1	0.7 ± 0.1
	Lean mass/ht^2^	19.6 ± 2.6	21 ± 2.7	16.2 ± 2.2	18.2 ± 2.3
	% ALM	0.2 ± 0	0.3 ± 0	0.3 ± 0	0.3 ± 0
	ALM/ht^2^	8.3 ± 1.4	9.1 ± 1.3	6.8 ± 1.2	7.9 ± 1.4
	Total lean mass	54 ± 11.8	58.9 ± 11.2	45.7 ± 9.9	52.3 ± 11.7
	Total fat mass	39.9 ± 9	37.4 ± 8.8	24 ± 6	24.1 ± 5
	% Fat mass	0.4 ± 0.1	0.4 ± 0.1	0.3 ± 0.1	0.3 ± 0.1
	Fat mass/ht^2^	14.8 ± 3.7	13.6 ± 3.6	8.7 ± 2.4	8.6 ± 2.1

Abbreviations: % ALM, appendicular lean mass per body weight; % fat mass, fat mass per body weight; % lean mass, total lean mass per body weight; ALM/ht^2^, appendicular lean mass per height squared; ALM, appendicular lean mass; NORC, Pennington/Louisiana Nutrition and Obesity Research Center.

1 Values are presented as mean ± SD.

**TABLE 2:** Test for sex differences in the association between body composition and HOMA-IR

Cohort	Body composition measure	BMI ≥30 kg/m^2^ (*P* value)	BMI <30 kg/m^2^ (*P* value)
NORC	% Lean mass	0.020^1^	0.567
	Lean mass/ht^2^	0.109	0.974
	% ALM	0.059	0.265
	ALM/ht^2^	0.011^1^	0.540
	Total lean mass	0.109	0.239
	Total fat mass	0.190	0.674
	% Fat mass	0.016^1^	0.224
	Fat mass/ht^2^	0.303	0.143
NHANES	% Lean mass	0.002^1^	0.083
	Lean mass/ht^2^	0.161	0.001^1^
	% ALM	0.043^1^	0.228
	ALM/ht^2^	0.267	0.005^1^
	Total lean mass	0.001^1^	0.068
	Total fat mass	0.653	0.110
	% Fat mass	0.002^1^	0.083
	Fat mass/ht^2^	0.047^1^	0.489

Abbreviations: % ALM, appendicular lean mass per body weight; % fat mass, fat mass per body weight; % lean mass, total lean mass per body weight; ALM/ht^2^, appendicular lean mass per height squared; NORC, Pennington/Louisiana Nutrition and Obesity Research Center.

1 Values are statistically significant (*P* < 0.05).

In the NORC cohort of older adults with obesity (*n* = 188), HOMA-IR positively correlated with total lean mass, lean mass/ht^2^, and ALM/ht^2^. However, this relationship was only observed in females. In males, % lean mass and % ALM negatively correlated with HOMA-IR, whereas % fat mass, total fat mass, and fat mass/ht^2^ positively correlated with HOMA-IR. Analysis of NHANES data using older adults with obesity (*n* = 756) showed similar positive relationships between total lean mass, lean mass/ht^2^, and ALM/ht^2^ with HOMA-IR, in females. Similar to the NORC cohort, these relationships were observed only in females. In males, % ALM negatively correlated with HOMA-IR, whereas % fat mass and fat mass/ht^2^ positively correlated with HOMA-IR. The results are presented in Table 3.

**TABLE 3:** Relationship between HOMA-IR and body composition measures in older adults with obesity (BMI ≥30 kg/m^2^)

NORC	Males (*n* = 82)				Females (*n* = 106)			
	*r*	95% confidence interval		*P* value	*r*	95% confidence interval		*P* value
% Lean mass	−0.220	−0.418	−0.002	0.048^1^	0.137	−0.056	0.320	0.164
Lean mass/ht^2^	−0.037	−0.253	0.183	0.744	0.301	0.116	0.466	0.002^1^
% ALM	−0.295	−0.483	−0.082	0.007^1^	0.035	−0.157	0.225	0.721
ALM/ht^2^	−0.155	−0.361	0.065	0.167	0.276	0.089	0.445	0.004^1^
Total lean mass	0.006	−0.213	0.224	0.961	0.270	0.082	0.439	0.005^1^
Total fat mass	0.265	0.049	0.457	0.017^1^	0.081	−0.112	0.269	0.410
% Fat mass	0.269	0.054	0.460	0.015^1^	−0.104	−0.290	0.090	0.293
Fat mass/ht^2^	0.254	0.038	0.448	0.022^1^	0.124	−0.069	0.308	0.208

**NHANES**	**Males (*****n*****= 327)**				**Females (*****n*****= 429)**			
	***r***	**95% confidence interval**		***P*****value**	***r***	**95% confidence interval**		***P*****value**
% Lean mass	−0.086	−0.197	0.026	0.132	0.094	**−**0.009	0.196	0.074
Lean mass/ht^2^	0.084	−0.026	0.194	0.136	0.200	0.102	0.299	<0.001^1^
% ALM	−0.121	−0.238	−0.003	0.044^1^	0.053	−0.044	0.151	0.285
ALM/ht^2^	0.030	−0.084	0.144	0.604	0.142	0.046	0.238	0.004^1^
Total lean mass	0.021	−0.089	0.130	0.708	0.229	0.131	0.326	<0.001^1^
Total fat mass	0.101	−0.009	0.210	0.071	0.080	−0.016	0.176	0.104
% Fat mass	0.115	0.004	0.225	0.043^1^	−0.098	−0.200	0.004	0.060
Fat mass/ht^2^	0.140	0.030	0.249	0.013^1^	0.043	−0.052	0.140	0.376

Abbreviations: % lean mass, total lean mass per body weight; %ALM, appendicular lean mass per body weight; ALM/ht^2^, appendicular lean mass per height squared; % fat mass, fat mass per body weight; *r*, Pearson correlation coefficient.

1 Values are statistically significant (*P* < 0.05).

In older adults without obesity (*n* = 113), the absolute measures of body composition, whether adjusted for weight or height, were not correlated with HOMA-IR in the NORC cohort. However, in the NHANES cohort, total lean mass, lean mass/ht^2^, and ALM/ht^2^ positively correlated with HOMA-IR in females. These relationships were not observed in males. In contrast, % lean mass and % ALM were negatively correlated, and fat mass and % fat mass were positively correlated with HOMA-IR in males. The results are presented in Table 4.

**TABLE 4:** Relationship between HOMA-IR and body composition measures in older adults with BMI <30 kg/m^2^ (without obesity)

NORC	Males (*n* = 57)				Females (*n* = 56)			
	*r*	95% confidence interval		*P* value	*r*	95% confidence interval		*P* value
% Lean mass	−0.087	−0.342	0.18	0.528	0.017	−0.25	0.281	0.903
Lean mass/ht^2^	0.021	−0.243	0.282	0.878	−0.062	−0.322	0.207	0.656
% ALM	−0.15	−0.397	0.118	0.272	0.086	−0.184	0.343	0.536
ALM/ht^2^	−0.06	−0.318	0.206	0.66	0.032	−0.236	0.294	0.82
Total lean mass	−0.128	−0.378	0.139	0.348	0.072	−0.197	0.331	0.604
Total fat mass	0.116	−0.152	0.368	0.396	0.046	−0.222	0.307	0.74
% Fat mass	0.206	−0.06	0.445	0.128	0.021	−0.245	0.285	0.878
Fat mass/ht^2^	0.09	−0.18	0.34	0.538	0.24	−0.02	0.47	0.072

**NHANES**	**Males (*****n*****= 717)**				**Females (*****n*****= 594)**			
	***r***	**95% Confidence interval**		***P*****value**	***r***	**95% Confidence interval**		***P*****value**
% Lean mass	−0.099	−0.175	−0.023	0.011^1^	−0.037	−0.119	0.046	0.383
Lean mass/ht^2^	0.058	−0.017	0.132	0.131	0.182	0.101	0.264	<0.001^1^
% ALM	−0.114	−0.189	−0.039	0.003^1^	−0.056	−0.137	0.026	0.18
ALM/ht^2^	0.017	−0.058	0.091	0.659	0.117	0.036	0.198	0.005^1^
Total lean mass	0.067	−0.008	0.141	0.079	0.139	0.058	0.22	0.001^1^
Total fat mass	0.126	0.052	0.201	0.001^1^	0.09	0.009	0.171	0.029^1^
% Fat mass	0.109	0.034	0.184	0.005^1^	0.032	−0.05	0.114	0.44
Fat mass/ht^2^	0.05	−0.02	0.13	0.154	0.17	0.09	0.25	<0.001^1^

Abbreviations: %ALM, appendicular lean mass per body weight; % fat mass, fat mass per body weight; % lean mass, total lean mass per body weight; ALM/ht^2^, appendicular lean mass per height squared; *r*, Pearson correlation coefficient.

1 Values are statistically significant (*P* < 0.05).

## Discussion

The aim of this study was to determine the relationship between insulin resistance and lean mass when reported as: *1*) % lean mass; *2*) lean mass/ht^2^; *3*) % ALM; and *4*) ALM/ht^2^ in older adults with and without obesity. Analysis of data from both the NORC and NHANES cohorts showed that total lean mass, lean mass/ht^2^, and ALM/ht^2^ correlated positively with HOMA-IR in females with obesity. In the NHANES cohort, this relationship of lean mass with HOMA-IR was evident in females with and without obesity. In males, % fat mass positively correlated with HOMA-IR, whereas lean mass did not appear to influence insulin resistance. Our results are consistent with prior studies showing that lean mass is positively associated with insulin resistance when adjusted for height [7,12]. However, an important finding of this study was that sexual dimorphism exists in the association of body composition with insulin resistance in older adults.

Skeletal muscle is the largest user of postprandial circulating glucose [19]. Yet, growing epidemiologic evidence suggests that lean mass may be associated with a less favorable glucose homeostasis. Lee et al. [5] found that in 5994 males aged ≥65 y, measurements of body weight and total lean, appendicular lean, total fat, and truncal fat mass were higher with increasing quartiles of HOMA-IR. Batsis et al. [9] conducted an analysis of NHANES data that included all adults aged ≥60 y. They found that absolute ALM and ALM/BMI positively correlated with HOMA-IR after adjusting for age, sex, race, education, smoking status, and arthritis. In another study of adults ranging in age from 60 to 86 y, the adjusted associations between log HOMA-IR and ALM were positive in participants who were either overweight or had obesity, and were significantly greater compared with those with normal BMI [6]. Similarly, in an analysis of NHANES data from adults aged 20 to 79 y, fat mass, lean mass, and ALM, and each of them normalized to height^2^, were positively associated with HOMA-IR. These relationships persisted among males and females [7]. In Pima Indians, obesity is associated with an increase in fat-free mass almost kilogram for kilogram with fat mass when compared with the lean state. This increased fat-free mass has been proposed to produce insulin resistance through obesity-induced biophysical changes such as altered capillary spacing in hypertrophied muscle cells [20].

Lagace et al. [8] determined the association between metabolic syndrome and lean mass when represented as lean mass, % lean mass, and lean mass/ht^2^ in adults aged from 50 to 79 y. They found a positive association of lean mass and lean mass/ht^2^ with the metabolic syndrome, but a negative association with % lean mass. Their conclusion was that the representations of lean mass significantly and strongly influence the direction of its association with the prevalence of metabolic syndrome. Representing lean mass as a percentage of body weight (% lean mass) could explain the role of fat mass rather than indicating the role of lean mass per se. In this population that did not strictly include participants with obesity, the findings of Lagace et al. are consistent with other studies [3,4]. However, in the other studies, the investigators chose to use % lean mass to form their conclusions because it did not depart from the commonly held belief that lean mass promotes glucose homeostasis [3,4]. Therefore, Lagace et al. [8,12] contend that using % lean mass to determine associations with insulin resistance will lead to flawed inferences.

In our analysis of 2 cohorts, we also found that the significance of the association between body composition and insulin sensitivity depended on sex. Adjustment of lean mass for height correlated positively with insulin resistance in females regardless of whether they had or did not have obesity. In males, the relationship between body composition and insulin resistance appears to be driven largely by fat mass (or % fat mass) in individuals with and without obesity.

Our results from NORC and NHANES cohorts show that the adjustment of lean mass to height is congruent with other studies described above, demonstrating that lean mass/ht^2^ may represent the role of lean mass in insulin resistance. However, from the sexual dimorphism in the relationship of body composition with insulin resistance that we observed, it appears that the influence of body mass (i.e., lean or fat mass) is sex-dependent in older adults.

Although in females, lean mass correlates strongly with insulin resistance, the proportion of fat mass plays a dominant role in males. In particular, abdominal fat, particularly visceral fat, is a risk factor for development of insulin resistance in both males and females [21]. Visceral fat is resistant to the actions of insulin, and the flow of fatty acids from visceral fat into portal circulation impairs several hepatic metabolic processes. Visceral adipose tissue is also a source of inflammatory cytokines that contribute to insulin resistance. Furthermore, excess visceral adiposity is effectively a marker of a lack of subcutaneous fat expandability [22]. Males tend to have central fat distribution (android or apple shape). Females tend to store fat peripherally (gynoid or pear shape), which is defined as fat deposited in the limbs and hips, particularly in the lower body [[23], [24], [25], [26]]. Peripheral fat distribution is associated with improved insulin sensitivity, compared with central deposition [27,28]. Moreover, visceral adiposity is positively correlated with hepatic fat accumulation, which leads to insulin resistance [29,30]. Differences in anatomical fat distribution may explain sexual dimorphism in the role of fat and lean mass in insulin resistance among older adults. These sex differences in body composition may also be influenced, at least in part, by sex hormones.

Testosterone and 17β-estradiol are central to the metabolic homeostasis of most cells in both sexes. When testosterone and 17β-estradiol production stop or decrease during aging, metabolic dysfunction develops [31]. Males have greater central adiposity than females [23]. The age-associated decline in sex hormones may exacerbate central adiposity in males and explain the strong correlation of fat mass with insulin resistance. In older adults, intermuscular adipose tissue increases by 18% each year [32]. Lean mass measured by DXA does not measure muscle mass directly. Total body lean mass measured by DXA includes tissue from organs such as the kidney and liver, intermuscular adipose tissue, as well as fibrotic and other lean tissue. Although males have larger musculature than females [23], lean mass/ht^2^ positively correlates with HOMA-IR in females but not in males. Intermuscular adipose tissue does not appear to be significantly different in males and females with metabolic syndrome [33]. Furthermore, intermuscular adipose tissue is not associated with metabolic syndrome in females [33]. Therefore, intermuscular adipose tissue may not explain the sexual dimorphism in the relationship between lean mass and insulin resistance. Studies quantifying skeletal muscle mass using methods such as MRI or creatine-(*methyl*-d_3_) dilution are needed to enhance our understanding of skeletal muscle biology and glucose metabolism [34].

Age-related changes in body mass vary between sexes. For instance, in the Healthy Aging and Body Composition study, overall, there was a small decline (0.3%–0.4%) in body mass over 2 y [35]. However, males lost a greater absolute amount of lean mass and gained more fat mass compared with females. The strong correlation between weight change and change in lean tissue observed in the study implies that the effect of an intervention developed to increase lean mass can only be interpreted correctly if changes in body weight are taken into account [35]. Alternatively, a measure that is less collinear with body weight, such as lean mass/ht^2^, is used for assessment.

Compared with a low-stable insulin resistance trajectory from early to middle adulthood, a high-increasing trajectory was linked to lower ALM normalized to BMI in middle-aged males, but not in females [36]. We observed a similar relationship in older adults. Thus, differences in body composition, fat distribution, and sex hormones may be relevant to sex differences we noted in the relationship between % lean mass and lean mass/ht^2^ and insulin resistance. Furthermore, it remains unclear whether the greater age-related decline in muscle mass observed in males compared with females results in more variability over time and contributes to the sex differences [35,36].

Importantly, weight loss interventions aimed at reducing excess fat often result in some degree of skeletal muscle loss. This loss may be exacerbated in individuals with underlying catabolic conditions (e.g., chronic disease or aging), or in the context of prolonged inadequate or unbalanced diets (particularly low protein intake), and repeated weight cycling [[37], [38], [39]]. Interestingly, our data suggest that sexual dimorphism in the relationship between body composition and insulin resistance is evident in older individuals with and without obesity. To optimize treatment strategies and assess long-term benefits, further research is needed to determine whether adjustment to body weight overestimates muscle mass and whether normalization by height may better reflect relative muscle mass.

Muscle fiber composition may also play a role: greater fat-free mass is typically linked to a higher proportion of type II (especially IIx) fibers, which have high hypertrophic potential but are inversely associated with insulin sensitivity [40]. This unfavorable metabolic profile is further exacerbated in older adults with metabolic syndrome due to reduced oxidative capacity and excess fat accumulation [12,41,42]. Consequently, sex-based differences in body composition may influence muscle fiber type distribution and the relationship between lean or fat mass and insulin resistance.

The main limitations of our study were that data on sex hormones and intermuscular adipose tissue were unavailable to support our analyses. Nevertheless, together with well-documented information on sex hormone concentrations and muscle fat infiltration in older males and females [[43], [44], [45], [46], [47], [48], [49]], the analysis provided new information on sexual dimorphism in metabolic regulation. In conclusion, the results of our study add to the growing consensus from epidemiologic studies indicating that increasing lean mass may not always promote insulin sensitivity. However, whether the observed association between lean mass and insulin resistance is because fat mass and lean mass both increase with weight gain, or whether it is due to a lack of understanding of skeletal muscle biology, remains unresolved. The contributions of muscle quality and sex hormones to insulin resistance in older adults warrant investigation in large randomized controlled trials.

## Author contributions

The authors’ responsibilities were as follows – CJR, SLSD, DZ, RAR: designed research, conducted research, reviewed and edited manuscript; FLG: designed research and reviewed and edited manuscript; CJR, DZ: wrote manuscript; CJR: primary responsible for final content; and all authors: read and approved the final manuscript.

## Data availability

The datasets generated and analyzed during the current study are not publicly available but are available from the corresponding author on reasonable request

## Declaration of Generative AI and AI-Assisted Technologies in the Writing Process

The authors declare that no generative AI or AI-assisted technologies were used in the writing of this manuscript.

## Funding

This work was supported in part by the by a grant from the National Institute on Aging (CJR, R00AG065419), by a NORC Center Grant # P30DK072476 titled “Nutrition and Metabolic Health Through the Lifespan” sponsored by the National Institute of Diabetes and Digestive and Kidney Diseases, and the National Institute of General Medical Sciences which funds the Louisiana Clinical and Translational Science Center (U54 GM104940). The content is solely the responsibility of the authors and does not necessarily represent the official views of the National Institutes of Health. All authors certify that they comply with the ethical guidelines for authorship and publishing in *Current Developments in Nutrition*.

## Conflict of interest

The authors report no conflicts of interest.
