# Supplementary material for: Sexual Dimorphism in the Relationship between Body Composition and Insulin Resistance in Older Adults
Source: Curr Dev Nutr. 2026 Apr 25;10(6):107707. doi: 10.1016/j.cdnut.2026.107707 (PMC13200084; doi:10.1016/j.cdnut.2026.107707)
Supplement: Multimedia component 1 [file mmc1.docx]

**Sexual Dimorphism in the Relationship Between Body Composition And Insulin Resistance in Older Adults**

Dachuan Zhang

**Supplementary Figure Legends:**

**Figure 1.** NHANES: Percent appendicular lean mass vs. HOMA‑IR by sex (body mass index ≥30 kg/m²). NHANES: National Health and Nutrition Examination Survey; BMI: body mass index; %ALM: appendicular lean mass/body weight; HOMA‑IR: Homeostatic Model Assessment of Insulin Resistance.

**Figure 2.** NHANES: Appendicular lean mass/height² vs. HOMA‑IR by sex (body mass index <30 kg/m²). NHANES: National Health and Nutrition Examination Survey; BMI: body mass index; ALM/ht²: appendicular lean mass/(height)²; HOMA‑IR: Homeostatic Model Assessment of Insulin Resistance.

**Figure 3.** NORC: Appendicular lean mass/height² vs. HOMA‑IR by sex (body mass index ≥30 kg/m²). NORC: Pennington/Louisiana Nutrition and Obesity Research Center; ALM/ht²: appendicular lean mass/(height)²; HOMA‑IR: Homeostatic Model Assessment of Insulin Resistance.

**Figure 4.** NHANES: Percent lean mass vs. HOMA‑IR by sex (body mass index ≥30 kg/m²). NHANES: National Health and Nutrition Examination Survey; BMI: body mass index; %lean mass: lean mass/body weight; HOMA‑IR: Homeostatic Model Assessment of Insulin Resistance.

**Figure 5.** NHANES: Lean mass/height² vs. HOMA‑IR by sex (body mass index <30 kg/m²). NHANES: National Health and Nutrition Examination Survey; BMI: body mass index; lean mass/ht²: lean mass/(height)²; HOMA‑IR: Homeostatic Model Assessment of Insulin Resistance.

**Figure 6.** NORC: Percent lean mass vs. HOMA‑IR by sex (body mass index ≥30 kg/m²). NORC: Pennington/Louisiana Nutrition and Obesity Research Center; %lean mass: lean mass/body weight; HOMA‑IR: Homeostatic Model Assessment of Insulin Resistance.

**Figure 7.** NHANES: Percent fat mass vs. HOMA‑IR by sex (body mass index ≥30 kg/m²). NHANES: National Health and Nutrition Examination Survey; BMI: body mass index; %fat mass: fat mass/body weight; HOMA‑IR: Homeostatic Model Assessment of Insulin Resistance.

**Figure 8.** NORC: Percent fat mass vs. HOMA‑IR by sex (body mass index ≥30 kg/m²). NORC: Pennington/Louisiana Nutrition and Obesity Research Center; %fat mass: fat mass/body weight; HOMA‑IR: Homeostatic Model Assessment of Insulin Resistance.

**Figure 9.** NHANES: Fat mass/height² vs. HOMA‑IR by sex (body mass index ≥30 kg/m²). NHANES: National Health and Nutrition Examination Survey; BMI: body mass index; fat mass/ht²: fat mass/(height)²; HOMA‑IR: Homeostatic Model Assessment of Insulin Resistance.

**Figure 10.** NHANES: Total lean mass vs. HOMA‑IR by sex (body mass index ≥30 kg/m²). NHANES: National Health and Nutrition Examination Survey; BMI: body mass index; HOMA‑IR: Homeostatic Model Assessment of Insulin Resistance.


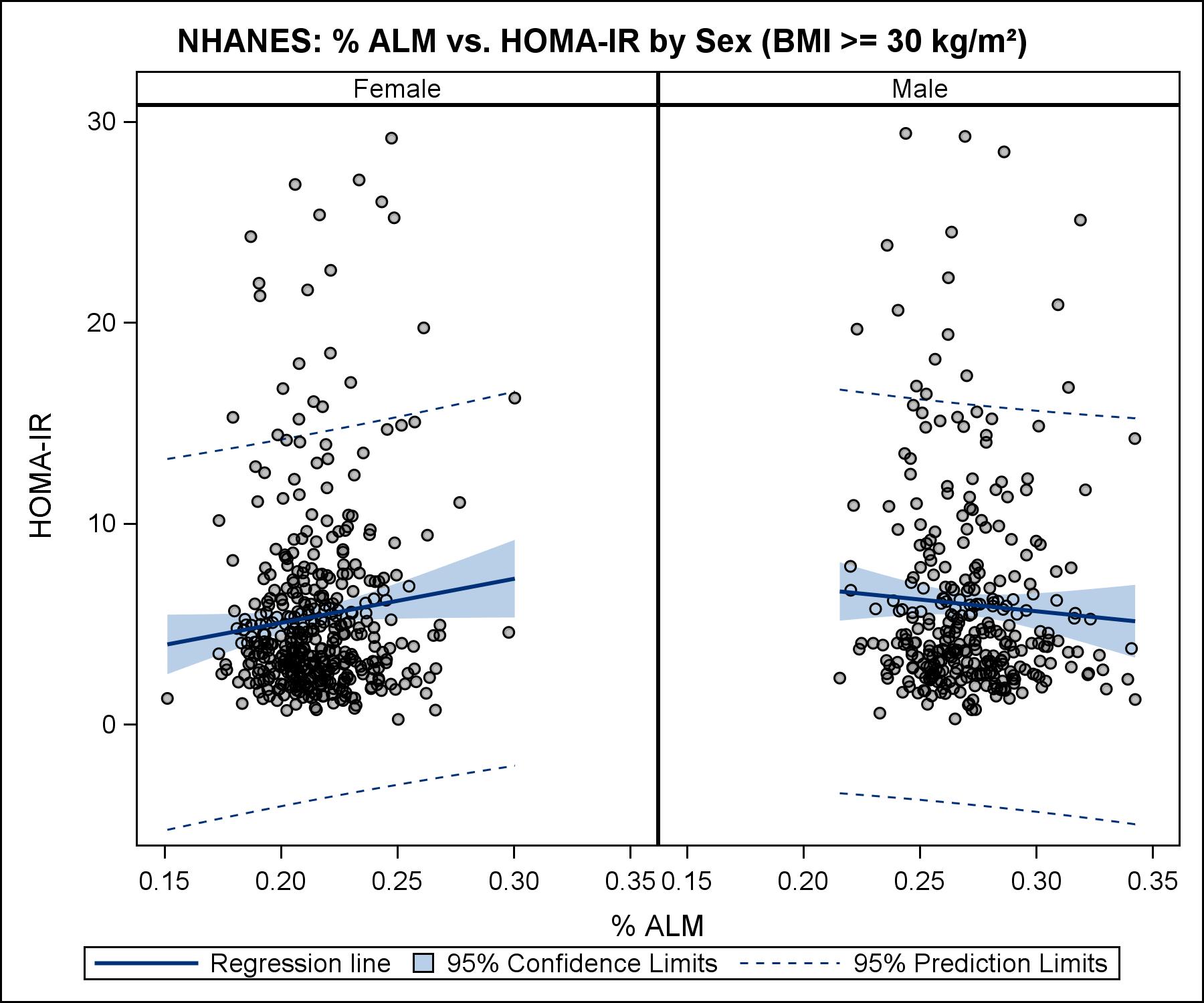


**Supplementary Figure 1.**


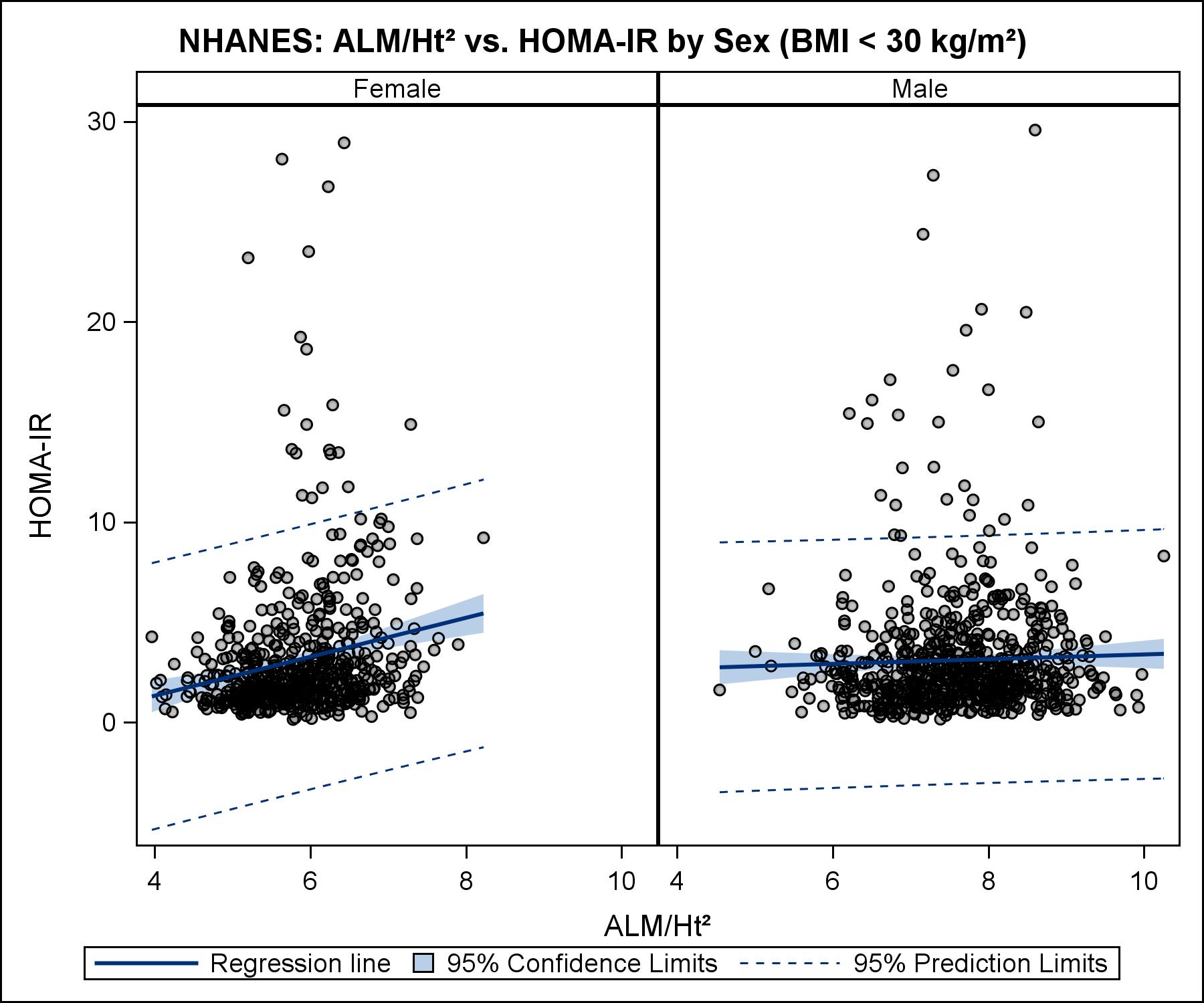
**Supplementary Figure 2.**


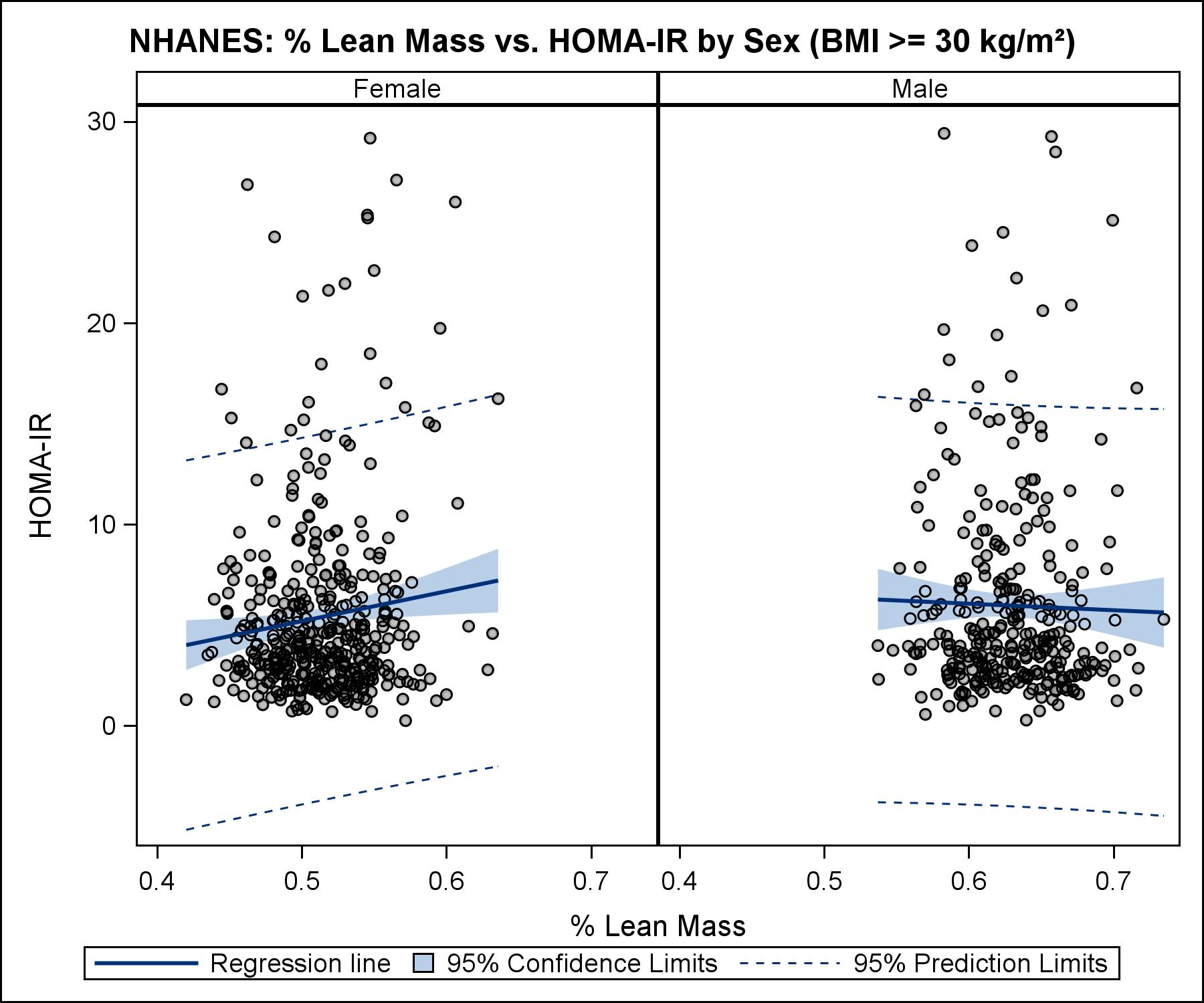


**Supplementary Figure 3.**


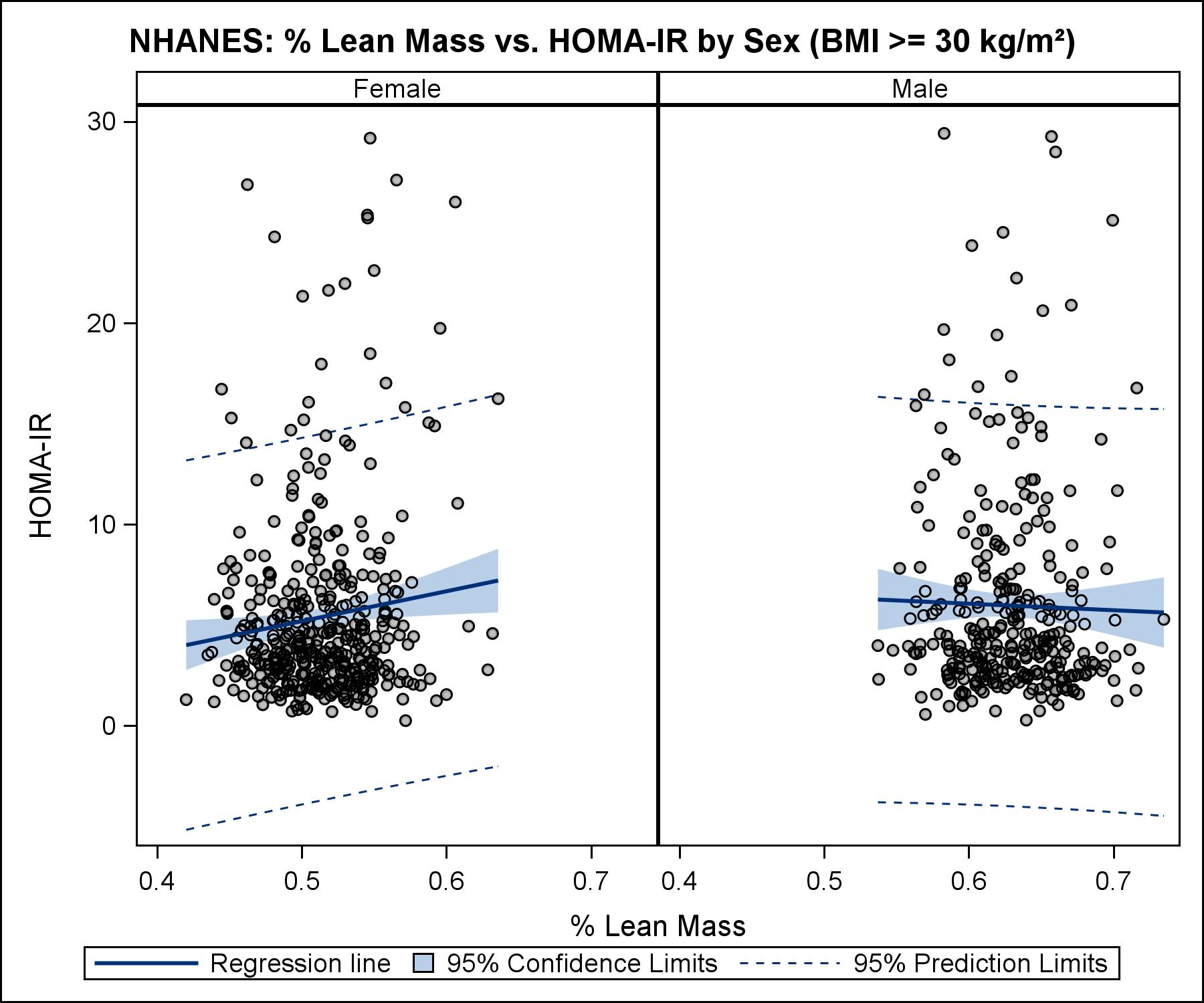


**Supplementary Figure 4.**


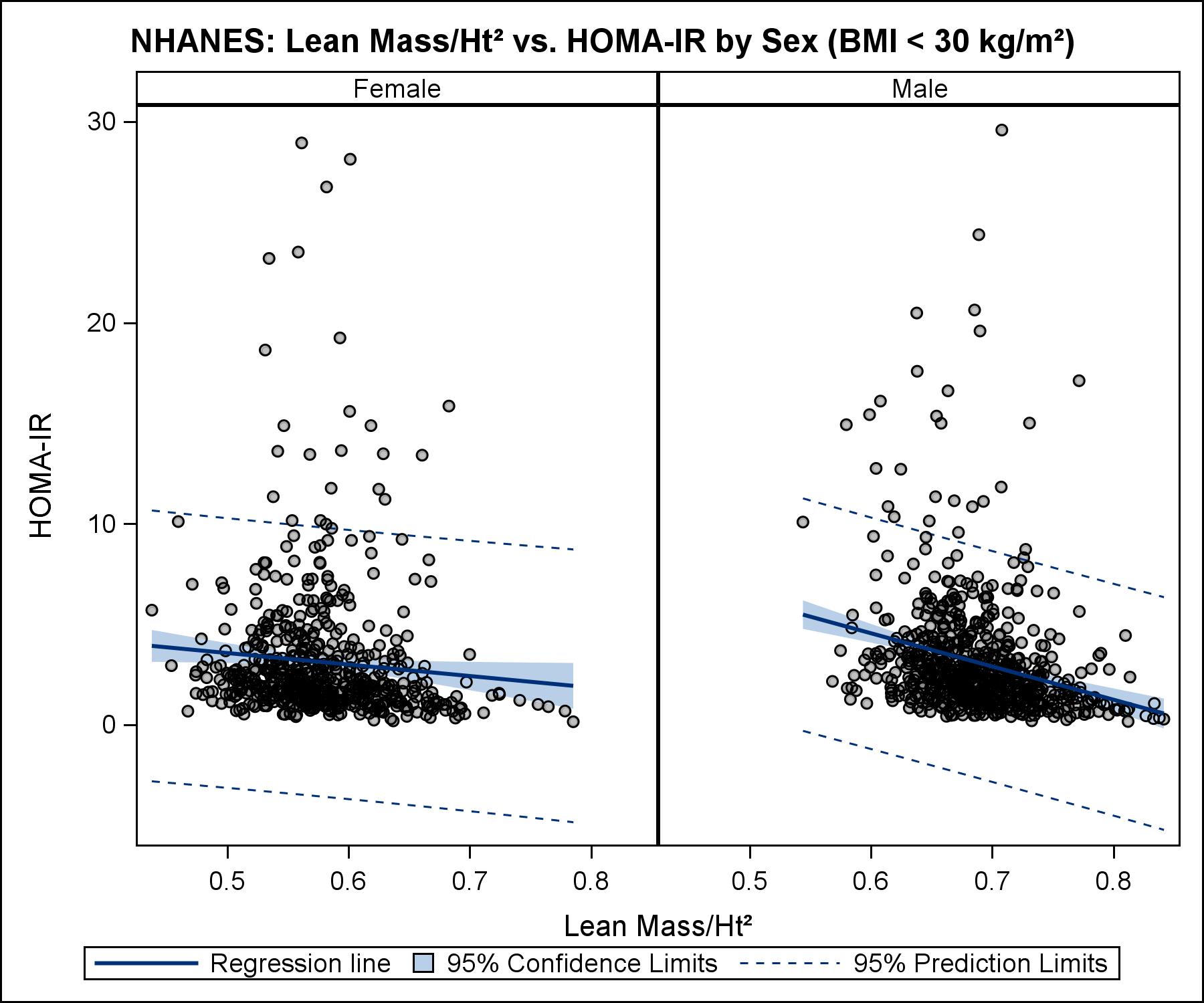


**Supplementary Figure 5.**


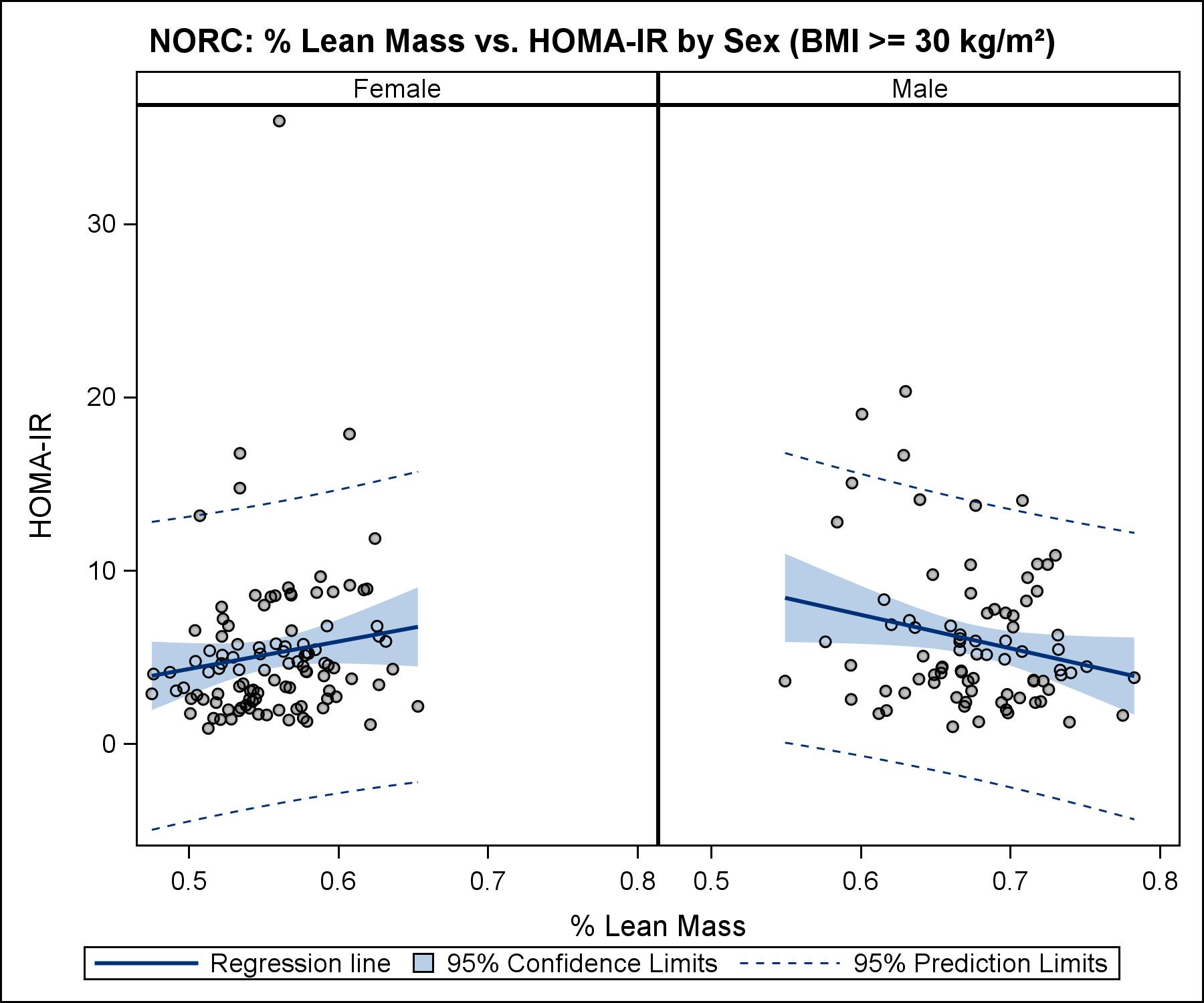


**Supplementary Figure 6.**


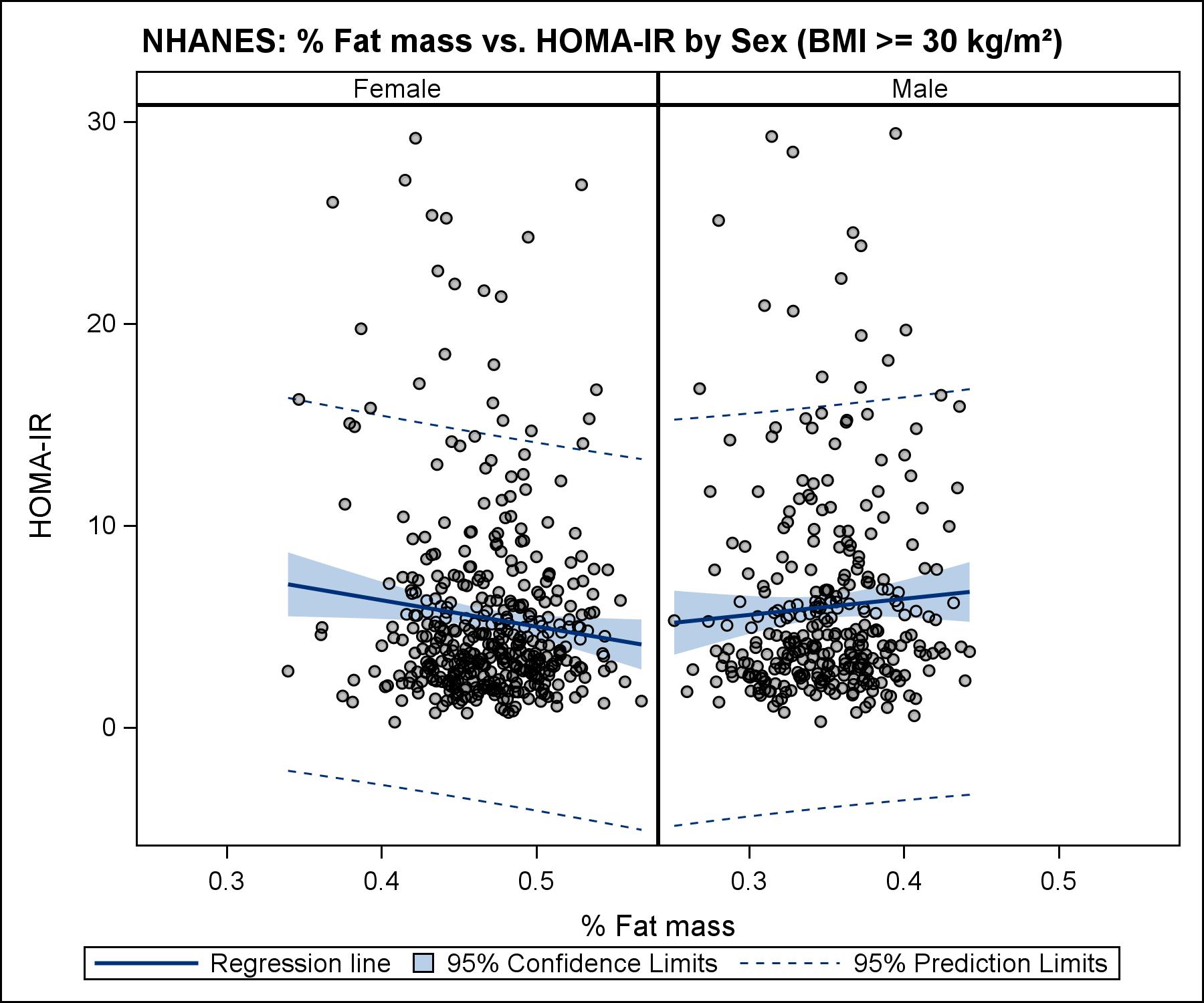


**Supplementary Figure 7.**


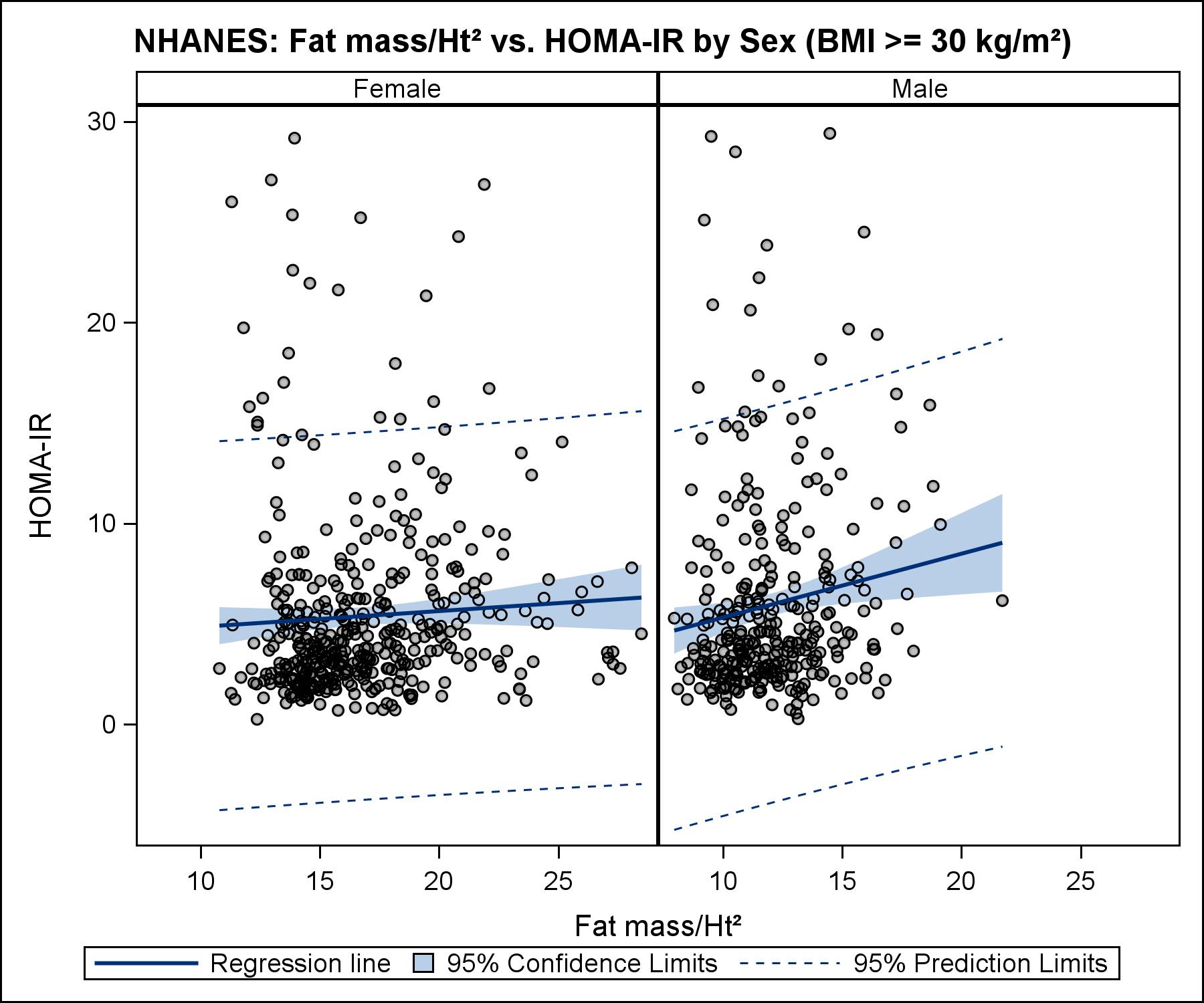


**Supplementary Figure 8.**


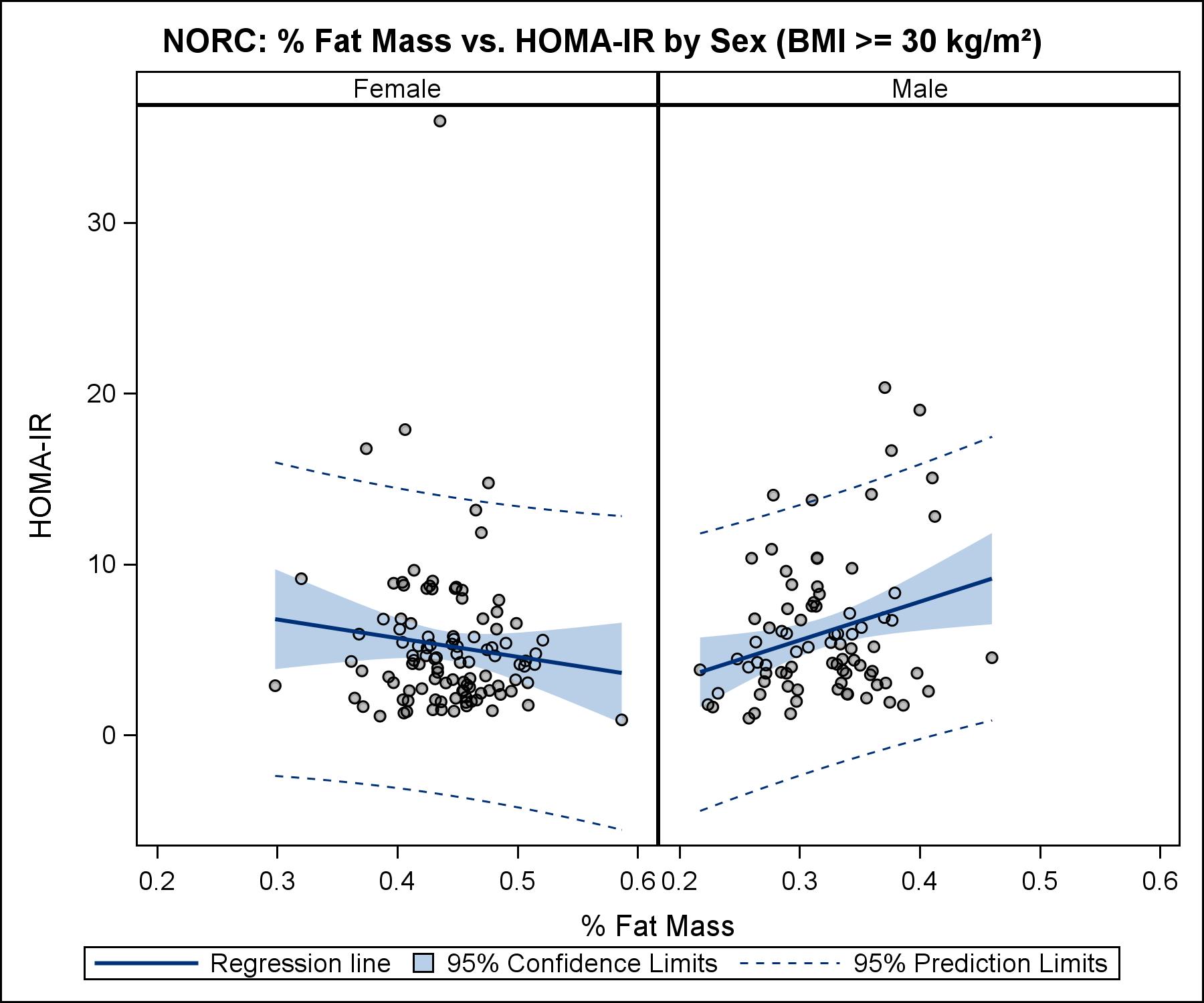


**Supplementary Figure 9.**


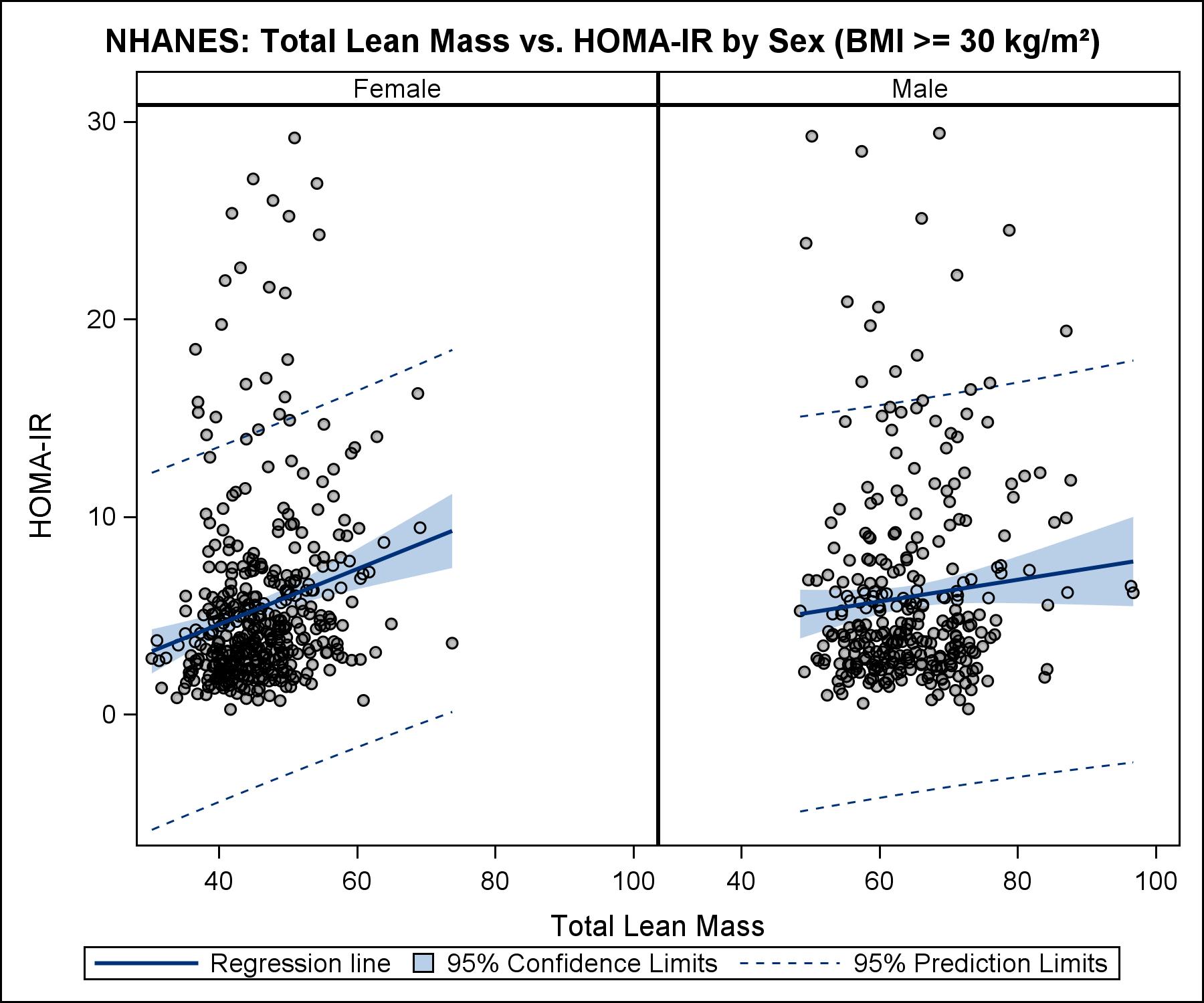


**Supplementary Figure 10.**
